# Children’s Blood Lead Concentrations from 1988 to 2015 in Mexico City: The Contribution of Lead in Air and Traditional Lead-Glazed Ceramics

**DOI:** 10.3390/ijerph15102153

**Published:** 2018-09-30

**Authors:** Ivan Pantic, Marcela Tamayo-Ortiz, Antonio Rosa-Parra, Luis Bautista-Arredondo, Robert O. Wright, Karen E. Peterson, Lourdes Schnaas, Stephen J. Rothenberg, Howard Hu, Martha María Téllez-Rojo

**Affiliations:** 1 Department of Developmental Neurobiology, National Institute of Perinatology, Mexico City 11000, CDMX, Mexico; ivandpantic@gmail.com (I.P.); lschnaas@hotmail.com (L.S.); 2 National Council of Science and Technology, Mexico City 03940, CDMX, Mexico; 3 Center for Nutrition and Health Research, National Institute of Public Health, Cuernavaca 62100, Morelos, Mexico; joseadlrp@gmail.com (A.R.-P.); lbautista@insp.mx (L.B.-A.); mmtellez@insp.mx (M.M.T.-R.); 4 Department of Environmental Medicine and Public Health, Icahn School of Medicine at Mount Sinai, New York, NY 10029, USA; robert.wright@mssm.edu; 5 Department of Nutritional Sciences, University of Michigan School of Public Health, Ann Arbor, MI 48109, USA; karenep@umich.edu; 6 Center for Population Health Research, National Institute of Public Health, Cuernavaca 62100, Morelos, Mexico; drlead@prodigy.net.mx; 7 Department of Environmental and Occupational Health Sciences, University of Washington School of Public Health, Seattle, WA 98195, USA; hhu5@uw.edu

**Keywords:** children’s blood lead, Mexico City cohorts, lead in air, lead-glazed ceramics

## Abstract

Despite the removal of lead from gasoline in 1997, elevated blood lead levels (BLLs) > 5 µg/dL are still detectable in children living in Mexico City. The use of lead-glazed ceramics may explain these persistent exposure levels. Mexico lacks a national surveillance program for BLL, but temporal trends can be derived from epidemiological studies. With this approach, we leveraged a series of birth cohorts to report BLL trends from 1987 to 2002 and expanded our analysis to 2015. Data were from 1–5-year-old children from five Mexico City cohorts followed between 1988 and 2015. BLLs are reported on 1963 children, who contributed 4975 BLLs. We estimated the trend of mean BLL, which decreased from 15.7 µg/dL in 1988, to 7.8 µg/dL in 1998 (a year after the total ban of lead in gasoline), to 1.96 µg/dL in 2015. The proportion of BLL ≥ 5 µg/dL decreased from 92% (1988–1998) to 8% (2008–2015). The use of lead-glazed ceramics was associated with an 11% increase in BLLs throughout the study period. Replacing lead-based glazes in traditional ceramics may be the key to further reducing exposure, but this presents challenges, as it involves a cultural tradition deeply rooted in Mexico. In addition, the creation of a rigorous, standardized, and on-going surveillance program of BLL is necessary for identifying vulnerable populations.

## 1. Introduction

The removal of lead from gasoline was one of the most important environmental public health measures of the last century and occurred in the majority of countries around the globe [1]. Mexico phased out lead from automobile gasoline, removing it completely in 1997 with a consequent marked reduction of lead levels in the air [2]. A study of 321 children by Schnaas et al. illustrated the parallel decline of concentrations of lead in air with children’s blood lead levels (BLL) between 1987 and 1998 [3]. Nevertheless, the use of traditional Mexican ceramics to prepare, serve, and store food has been identified as an important source of lead exposure for centuries and still persists [4,5]. The final coating of these ceramics is a lead monoxide glaze, the low melting point of which makes it ideal for use in low-temperature (<800 °C) wood-fired kilns. When in contact with food, lead will leach from the vessel [6]. Lead-glazed traditional ceramics (PbC) are embedded in Mexican culture and used throughout the country. Lead has been widely documented as a neurotoxicant in children with effects that can last into adulthood [7], even at levels below the U.S. Centers for Disease Control and Prevention’s (CDC) reference BLL and the current Mexican reference value of 5 µg/dL [8,9]. Lead has also been conclusively linked to the causation of hypertension and other cardiovascular diseases in adults, with a recent study by Lanphear et al. generating an estimate of 18.0% (95% CI: 10.9–26.1) for the attributable fraction of BLL on all-cause mortality for U.S. adults, which is almost half a million annual deaths. In that study, the geometric mean for BLL was 2.7 µg/dL [10]. A meta-analysis using the results from 83 articles from Mexico, conducted by Caravanos et al., reports a geometric mean of BLL for Mexican children and adults of urban populations of 8.8 µg/dL in the period between 1978 and 2010 and a 5.4-µg/dL geometric mean BLL since the phase-out of leaded gasoline [11]. These types of analyses using epidemiologic data allow the estimation of spatial and temporal trends in BLLs in Mexico, since a monitoring program of BLLs is lacking.

In contrast, the National Health Nutrition and Examination Surveys of the United States (NHANES) routinely measures BLL in population-wide representative surveys of the U.S. population, and in a recent article, Tsoi et al. documented the decline between 1999 and 2014: the mean (95% CI) descended from 1.65 µg/dL (1.62–1.68) in 1999–2000 to 0.84 µg/dL (0.82–0.86) in 2013–2014. They reported that the percentage of 1–5-year-old children with BLL > 5 µg/dL decreased from 9.9% (7.5–12.9) in 1999–2000 to 0.5% (0.3–1.0) in 2013–2014 [12]. In 2015 in Mexico, Téllez-Rojo et al. carried out a cross-sectional study of BLL in cord blood of 300 newborns from the state of Morelos. Although the mean BLL was 3.1 µg/dL (2.7–3.5), the prevalence of children with BLL > 5 µg/dL was 14.7% (95% CI: 11.1–19.3). It was also found that newborns whose mothers did not use lead-glazed traditional ceramics during pregnancy had a mean cord BLL of 1.3 µg/dL (95% CI: 1.1–1.5), whereas newborns whose mothers reported using lead-glazed ceramics had BLL of 3.5 µg/dL (95% CI: 2.4–5.0) [13]. As can be noted from the results of the studies mentioned above, focusing only on the mean BLL in relation to the reference value of 5 µg/dL might suggest that lead exposure is not a problem. However, the prevalence of children with levels above this reference level provides a more informative estimate of the elevated BLLs among children and may help to identify undetected lead exposure sources. 

Our research group has been studying the effects of early life exposure to lead in Mexico City for more than three decades. We collected data on children’s BLLs and the use of PbC for five cohorts between 1987 and 2015. The oldest of these cohorts was that which was reported by Schnaas et al. in 2004, which included data from 1988 to 1998. The aim of our present study was to expand the information from this first cohort using subsequent cohorts to illustrate the descent of BLL in 1–5-year-old children from 1987 to 2015. We included data on Pb in air, which we hypothesized would be associated with a parallel decrease in BLL. We also aimed to analyze the use of PbC throughout the study period in order to illustrate its past and current contribution to the BLLs of children. We hypothesized that the proportion of women reporting its use as well as its contribution to BLL would be higher than 5% throughout the study period.

## 2. Methods

We merged data from 1–5-year-old children from five birth cohorts from Mexico City. Cohort A was recruited in 1987 at the National Institute of Perinatology (INPer); cohorts B, C, D, and E were recruited in clinics belonging to the Mexican Social Security System. Recruitment of participants started in 1994, 1997, 2001, and 2007 for cohorts B, C, D, and E, respectively. Cohorts B, C, and D are part of the Early Life Exposure in Mexico to ENvironmental Toxicants (ELEMENT) Project, and cohort E is part of the Programming Research in Obesity, Growth, Environment and Social Stressors (PROGRESS) project. All cohorts originally included healthy pregnant women planning to live in Mexico City for at least 2 years and to give birth at either INPer (cohort A) or at Gineco4 (cohorts B–E). Details on each of the cohorts can be found in previously published articles [3,14,15,16,17,18,19]. We included all available children’s BLLs measured in venous blood samples taken at 1, 2, 3, 4, and 5 years of age. Blood samples were analyzed by anodic stripping voltammetry (samples ≥ 5 μg/dL) and graphite furnace atomic absorption spectrometry (samples < 5 μg/dL) (cohort A), graphite furnace atomic absorption spectrophotometry (cohorts B and C), and inductively coupled plasma mass spectrometry (cohorts C, D, and E). Every BLL in our analyses was dated by the year in which it was collected. 

Information on the mean concentration of lead in air was calculated based on publicly available data downloaded from the Environment Ministry’s air pollution website (SIMAT) [20]. The number of monitoring stations distributed throughout Mexico City ranged from 9 to 19, from 8 January 1989 to 30 December 2013. Lead in air was measured in total suspended particles (TSP) every 6 days throughout the year. We averaged all the available measures for each calendar year and assigned the mean concentration to the BLLs collected that particular year. The measurement of lead in air stopped in 2013, so we assumed concentrations did not change for 2014 and 2015 and included them as such in our analysis. 

Information on current use of PbC was obtained through a questionnaire administered to the child’s mother during pregnancy. Since each cohort collected this information at different time points during pregnancy, we used the first available data (i.e., for some cohorts, data is from the first trimester and for others in the second trimester). Information on child’s sex and maternal school education was obtained from study questionnaires. Maternal school education was used as a proxy for socioeconomic status and was used as a categorical variable (elementary, technical middle school, middle school, technical high school, and high school or college). 

We used a mixed effect model with the natural log of BLL as the dependent variable (all BLLs available were introduced as the single outcome variable). The model was adjusted for use of PbC during pregnancy (yes/no), cohort, year in which the BLL was measured, child’s sex, maternal education, and an interaction term between average annual lead in air and age at sample collection (an ordinal variable 1, 2, 3, 4, and 5 years). A random intercept was included for each subject along with the random slope of BLL across the first 5 years of life utilizing an unstructured covariance matrix to account for within subject variance across the first 5 years of life. In order to examine if the relationship between use of PbC and BLL changed before and after the removal of lead from gasoline, we ran an additional model with an interaction term between PbC and an indicator variable before or after 1998. 

Finally, in order to explore the contribution of lead in air at each stage of the children’s development, we graphed the effect of lead in air and child’s age using the results of the model and contrasted the average marginal effects of each age. We used predictive margins to calculate the effect of calendar year on children’s BLLs and graphed the results along with the annual lead air concentration.

## 3. Results

Our study covered over 20 years of recruitment of pregnant women in five cohorts, spanning from 1987 to 2008, and 27 years of children’s BLLs (measured from 1988 to 2015). In total, 1963 children provided 4975 blood samples at different stages of the cohorts that correspond to children’s ages of 1, 2, 3, 4, and 5 years. For cohort A, children provided on average four blood samples; cohort B: three blood samples; cohort C: four blood samples; cohort D: three blood samples. For cohorts A–D, the samples collected were min = 1, max = 5; cohort E children provided on average two blood samples, min = 1, max = 3. Table 1 summarizes the characteristics of the study participants as well as the mean air lead concentrations and percentage of PbC use for each cohort. Participants’ reported use of PbC was most frequent in the two oldest cohorts: 41% in cohort A and 47% in cohort B; however, a third of the women reported using PbC during pregnancy in cohorts D and E. As expected, the mean (SD) air lead concentrations decreased from 0.56 µg/m^3^ (0.41) for cohort A (1988–1998) to 0.03 µg/m^3^ (0.00) for cohort E (2008–2015). That trend was matched by a reduction of mean blood lead concentrations over the cohorts, which is summarized in Table 2, stratified by children’s age. In cohort A, the geometric mean (SD) BLL was 9.5 µg/dL (1.7), cohort B = 7.2 µg/dL (1.6), cohort C = 4.8 µg/dL (1.8), and cohorts D and E = 4.0 (1.7) and 2.0 µg/dL (1.8), respectively. 

**Figure 1:**
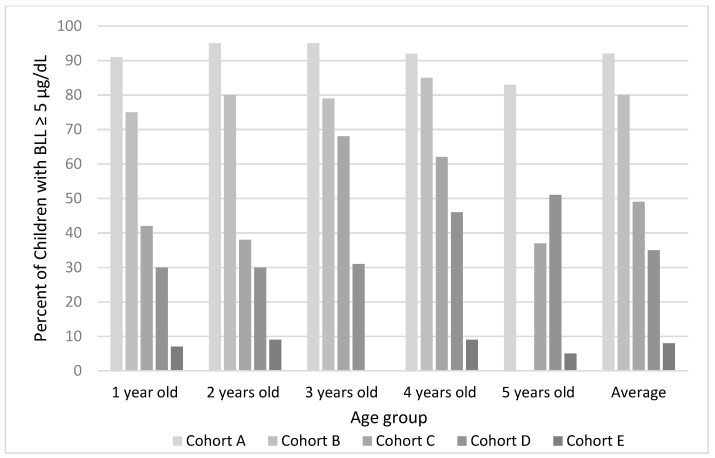
Percent of children with lead in blood concentrations higher than 5 µg/dL for each age group and cohort.

The percent of children with BLL higher than 5 µg/dL decreased significantly as well, from the oldest to the most recent cohort. In cohort A, 92% of children had BLL ≥ 5 µg/dL, whereas only 8% in cohort E had blood lead concentrations over that reference value. Figure 1 shows the change for each cohort stratified by children’s age.

**Figure 2:**
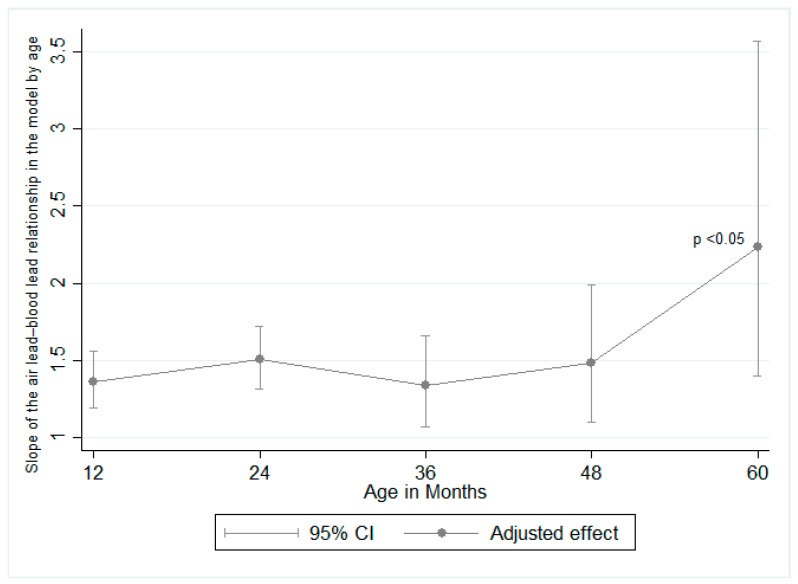
Contribution of lead in air on blood lead levels at each age. The 5-year slope of air lead on blood lead was significantly higher than the 1-year slope.

**Figure 3:**
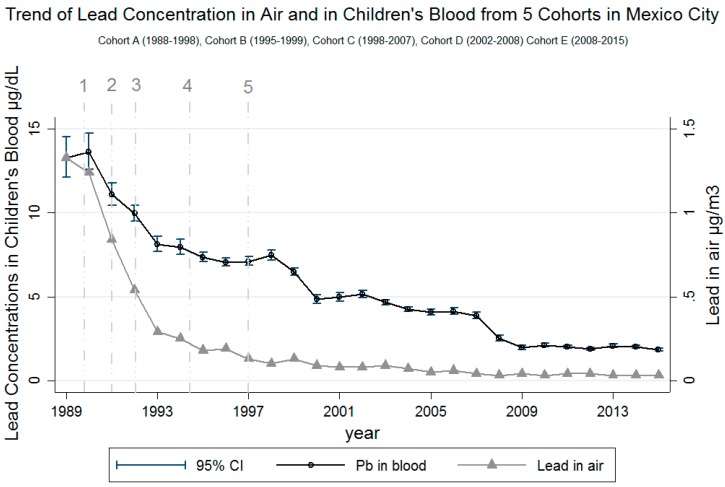
Results of adjusted mixed models for blood lead concentrations and air lead concentrations over the years. 1 = 1990: Introduction of “Magna Sin” (reduced lead) gasoline and one day a week without driving your car program. 2 = 1991: Closure of oil refinery in Mexico City (Azcapotzalco). Reduction to 0.5–1 mL/gal of tetraethyl lead in gasoline. 3 = 1992: Reduction to 0.2–0.3 mL/gal of tetraethyl lead in gasoline. 4 = 1994: Reduction to 0.1–0.2 mL/gal of tetraethyl lead in gasoline. 5 = 1997: Lead completely phased out of gasoline. 2014 and 2015 air lead levels were extrapolated from the 2013 air lead levels due to lead in air no longer being measured after 2013.

Results from the mixed effects model show that children of women who reported not using PbC had 11% (95% CI: 16%, 7%) lower BLL than children of women who reported using PbC (Table 3). This was consistent across the study period. The interaction between use of PbC and year of lead removal from gasoline did not yield different information; therefore, it was left out of the final model. As for the interaction between the child’s age and lead concentrations in air, the results of the contrasts of the marginal linear predictions yielded a statistically significant result (*p* value = 0.006), indicating that the contribution of lead in air to children’s BLLs varies by age and is the strongest at age 5. The results of these analyses are illustrated in Figure 2. Each 1-year increment in which the blood sample was taken was associated with a 7% mean decrease in BLL (95% CI: 9%, 5%). This is illustrated in Figure 3, where there is a clear decreasing trend in BLL over the entire study period. This figure also illustrates the trends in lead in air and BLL over the years for all the cohorts. The content of lead permitted in gasoline was gradually reduced starting in 1988 until it was completely eliminated in 1997 [2]. This was parallel to the reduction of mean air lead concentrations, from >1 µg/m^3^ in 1988 to <0.03 µg/m^3^ by 2013. 

Absence of information on covariates accounted for missing data that was not included in the model (4888 observations from 1892 children). This was mostly from missing air lead concentrations—as some of the first samples in cohort A were taken before lead was measured in air—and missing information on use of PbC or maternal education.

## 4. Discussion

Our results show that BLLs and air lead concentrations have continued to decline throughout the length of the study into 2015. The complete removal of lead from gasoline in 1997 likely has played a major role, although there is still work to be done to lower children’s BLL further, as pockets of elevated BLL remain. These results are in line with the trend previously found by Schnass et al. in their study [3]. The decline of mean lead concentrations in air and blood are clearly illustrated both by cohort (Table 1) as well as by calendar year (Figure 2). This trend is consistent with the NHANES data [12]; however, mean BLLs observed in our study were much higher. The BLL for 1–5-year-old children in the United States that is within the time period reported in our study was reported in the 1988–1991 NHANES survey: 3.6 µg/dL (95% CI: 3.3, 4.0) compared to 9.5 (SD 1.7) µg/dL in cohort A of our study. The lowest mean BLL in our study was 2.0 µg/dL (SD 1.8) for cohort E (2008–2015), whereas the NHANES 2013–2014 reported 0.84 μg/dL (95% CI, 0.82–0.86) [12]. Moreover, in our study, 8% (95% CI: 6.2, 11.4) of children had elevated BLLs (≥5 µg/dL) in cohort E (2008–2015) compared to 0.5% (95% CI, 0.3, 1.0) in the 2013–2014 survey. To further contextualize this, in the recent case of exposure to lead from drinking water in Flint, Michigan, the proportion of children with BLLs over the reference level increased from 2.4% to 4.9% [21]. These comparisons are of particular relevance in the context of possible sources of exposure to lead in Mexico, where an identified source is the use of PbC.

Our study found that children whose mothers reported not using PbC had 11% lower BLLs than those whose mothers reported using them. These results were consistent throughout the study period and the model was adjusted for maternal education, child’s sex, cohort, and lead in air. Children in this study belong to cohorts, the main objective of which, was to study the effect of lead exposure on neurodevelopment. As such, information on lead exposure and specifically on the use of PbC was provided to study participants. Despite this, women reported continued use of these ceramics. If, in fact, there is under-reporting of their use, our results could be underestimated. The use of PbC persists in Mexico since it is deeply embedded in the culture, and while there have been multiple success stories of programs to enable artisans to change their production to lead-free glazes [22,23], this remains a problem. There is a Federal law that prohibits the use of lead in consumer products, specifically, traditional ceramics (NOM-231-SSA1-2002). However, its enforcement is a matter that crosses multiple layers of politics of both private and public sectors [24].

In Mexico, data on BLL come mainly from epidemiologic studies. A recent meta-analysis included data from Mexico, but the studies were mostly of samples with point exposure sources (for example, smelters, occupational exposure) and included data only from cohort A from our study. Nonetheless, lead-glazed ceramics was identified as a source of exposure in most studies [25]. 

Despite the mean air lead concentration in 2013 being below Mexican quality standards (0.15 µg/m^3^ in TSP as a 3-month average) [20], our model suggests an important contribution of lead in air to BLL, which is accentuated for 5-year-old children compared to 1-year-olds (Figure 1). When looking at the figure, it is important to recall that the interaction term between lead in air and child’s age was statistically significant, since the broadly overlapping CIs in the figure could be misleading. These results were adjusted for sex, year, use of lead-glazed ceramics, and maternal education (as a proxy for SES) in the mixed model. This could indicate that younger children spend less time outside and therefore are less exposed to Pb in air. Although the results of the model show that lead in air has the strongest contribution to 5-year-olds’ BLLs, they have the lowest BLL geometric mean of 3.6 (2.2) µg/dL (Table 2). We hypothesize that other sources of lead exposure contribute more significantly than air to increase younger children’s BLLs since they have a different behavior (crawling and hand-to-mouth activity).

We are aware that our study did not account for a finer measure of Pb in air. We used the mean annual lead concentration for air for all air monitors in the city as the proxy for lead in air exposure for the children. This is a strong assumption since Mexico City covers 1485 km² and has geographic and meteorological conditions that could yield different concentrations of lead in air throughout the year and across the city. However, we believe it is unlikely that our results could be overestimated since our study participants were not clustered in a specific area of the city. Having more precise estimates would reduce the measurement error and likely strengthen the association found. We did not account for seasonality; indeed, a study by Haley and Talbot found that blood lead concentrations were higher in the summer compared to those collected in winter [26]. However, seasonality is different in Mexico City, which has a cold-rainy, a cold-dry, and a hot-dry season. Accounting for seasonality was beyond the objective of the study and including this information would require including data on lead concentrations in air specific to the exact BLL collection date. In order to evaluate if lead in air is still contributing to children’s BLLs, a more specific exposure assessment would be required. 

Throughout the follow-up of each cohort, study participants were provided with information on the use of PbC and the health effects of exposure to lead. To avoid response bias, our analyses included the first reported use of PbC, which was acquired during pregnancy. The fact that BLLs remain elevated in childhood suggests that PbC use continues postpartum, but there may be possible unknown sources of lead exposure. 

Eliminating lead exposure should be a priority for all countries since there is no safe blood lead level [27]. The immediate objective of our study is to add to the evidence of the success of the reduction of lead in air and its reflection in children’s BLLs, and its long-term objective is to highlight PbC as an identified source of lead exposure and the evidence of its contribution to children’s BLLs in order to help policy enforcement.

## 5. Conclusions

Twenty years after eliminating leaded gasoline, remarkable progress has been made in reducing childhood lead poisoning. While this is a public health success, the work is not complete, as the proportion of children 1–5-years old in Mexico City who exceed the U.S. CDC reference level remains substantial. Reporting only mean BLL can be misleading and is an incomplete assessment of the picture, as 8% of children were >5 µg/dL, representing approximately 51,000 children in Mexico City alone [28]. A study assessing personal exposure to lead in air could provide useful updated information on air-borne exposure sources. However, the use of lead-glazed ceramics was confirmed as an important contributor of children’s BLLs. Home assessments in future studies, including food, water, dust, and air, may also illuminate other sources of Pb exposure. 

## Figures and Tables

**Table 1:** Characteristics of study participants.

Characteristic	Cohort					Total
	A	B	C	D	E	
Recruitment start year	1987	1994	1997	2001	2007	
Years included in study	1988–1998	1995–1999	1998–2007	2002–2008	2008–2015	
Number of participants	291	457	240	382	593	1963
Girls, *n* (%)	129 (44)	202 (44)	122 (51)	190 (50)	293 (49)	936 (48)
	**Maternal Education, *n* (%)**					
Primary	55 (19)	94 (21)	27 (11)	28 (7)	17 (3)	219 (11)
Technical secondary	107 (38)	239 (53)	113 (47)	212 (56)	229 (39)	900 (46)
Secondary	74 (26)	69 (15)	68 (29)	89 (23)	212 (36)	512 (26)
Technical high school	42 (15)	43 (10)	27 (11)	49 (13)	128 (22)	289 (15)
High school or college	3 (1)	7 (1)	4 (2)	2 (1)	7 (1)	23 (2)
	**Number of Blood Samples for Each Age**					
1 year old	247	212	199	268	165	1091
2 years old	219	306	206	318	245	1294
3 years old	191	228	179	251	-	849
4 years old	185	263	194	188	251	1081
5 years old	151	-	184	80	245	660
Total number of samples	993	1009	962	1105	906	4975
**Main Exposures**						
Pb in air, mean (µg/m^3^), (SD) *	0.56 (0.41)	0.14 (0.04)	0.08 (0.01)	0.06 (0.01)	0.03 (0.00)	
Use of Pb-glazed ceramics, yes (%)	115 (41)	200 (47)	82 (35)	88 (23)	193 (33)	678 (36)

Note: Percentages may not add up to exactly 100%, owing to the rounding off; * Pb in air was measured from 1988 to 2013.

**Table 2:** Geometric mean (GM) for concentrations of lead in blood for each age group and cohort.

Age	Cohort					Total
	A	B	C	D	E	
	1988–1998	1995–1999	1998–2007	2002–2008	2008–2015	
GM (SD) (µg/dL)						
1 year old	9.9 (1.8)	6.7 (1.6)	4.3 (1.9)	3.6 (1.8)	2.2 (1.8)	4.9 (2.2)
2 years old	10.7 (1.7)	7.3 (1.6)	4.1 (1.9)	3.9 (1.8)	2.4 (1.7)	4.9 (2.1)
3 years old	9.9 (1.7)	7.3 (1.7)	6.2 (1.7)	4.0 (1.6)	-	6.3 (1.8)
4 years old	8.9 (1.6)	7.4 (1.5)	5.8 (1.6)	4.8 (1.7)	1.9 (1.9)	5.0 (2.1)
5 years old	7.8 (1.7)	-	4.4 (1.6)	4.9 (1.4)	1.8 (1.8)	3.6 (2.2)
GM (SD)	9.5 (1.7)	7.2 (1.6)	4.8 (1.8)	4.0 (1.7)	2.0 (1.8)	4.9 (2.1)

**Table 3:** Results of the mixed model for the log of children’s blood lead concentrations.

Variable	Coefficient	Standard Error	95% Confidence Interval
No use of Pb-glazed ceramics	−0.11	0.02	−0.15, −0.07 ***
Lead in air	0.32	0.07	0.18, 0.44 ***
Child’s age			
2 years old	0.11	0.02	0.07, 0.16 ***
3 years old	0.28	0.03	0.21, 0.35 ***
4 years old	0.33	0.05	0.24, 0.42 ***
5 years old	0.19	0.06	0.08, 0.31 ***
Lead in air × child’s age			
2 years old	0.09	0.05	−0.01, 0.20 *
3 years old	−0.02	0.09	−0.19, 0.15
4 years old	0.08	0.12	−0.15, 0.31
5 years old	0.49	0.20	0.09, 0.89 **
Year of sample collection	−0.07	0.01	−0.09, −0.05 ***
Cohort			
Cohort B	0.17	0.05	0.06, 0.28 **
Cohort C	0.18	0.10	−0.02, 0.37 *
Cohort D	0.27	0.14	−0.00, 0.53 *
Cohort E	0.11	0.22	−0.32, 0.53
Maternal education			
Elementary	0.32	0.27	−0.21, 0.86
Technical elementary	0.27	0.27	−0.26, 0.80
Middle school	0.22	0.27	−0.31, 0.75
Technical middle school	0.07	0.27	−0.46, 0.61
Highs school or college	−0.09	0.29	−0.67, 0.47
Sex (female)	−0.03	0.02	−0.07, 0.01

*** *p* value < 0.001, ** *p* value < 0.05, * *p* value < 0.1.
